# Supplementary material for: Association of Serum Levels and Immunohistochemical Labelling of Des-Gamma-Carboxy-Prothrombin in Patients Undergoing Liver Transplantation for Hepatocellular Carcinoma
Source: Diagnostics (Basel). 2024 Apr 25;14(9):894. doi: 10.3390/diagnostics14090894 (PMC11083058; doi:10.3390/diagnostics14090894)
Supplement: Supplementary file 1 [file diagnostics-14-00894-s001.zip › diagnostics-2914550-supplementary.pdf]

Supplementary Table S1. Characteristics of liver transplant recipients per intensity of des-γ-carboxy prothrombin labelling of hepatocellular carcinoma.[a]

	Whole population (n=39)	Negative labelling (n=13)	Focally positive labelling (n=7)	Diffusely positive labelling (n=19)	p[b]
	Medians (IQR) or n (%)				
Lab panel at LT					
Albumin (g/l)	37 (31-44)	44 (38-46)	36 (29-42)	36 (31-39)	0.060
Platelets (*10^3/ml)	106 (72-151)	100 (88-118)	124 (86-165)	112 (60-166)	0.638
Total bilirubin (mg/dl)	1.0 (0.7-2.3)	0.8 (0.5-2.0)	1.0 (0.6-3.8)	1.1 (0.9-2.3)	0.502
AST (U/l)	42 (31-77)	37 (28-61)	39 (32-70)	45 (37-81)	0.288
ALT (U/l)	33 (22-45)	36 (27-44)	31 (24-35)	28 (22-64)	0.808
INR	1.23 (1.09-1.39)	1.13 (1.06-1.27)	1.14 (1.09-1.76)	1.30 (1.13-1.60)	0.097
Creatinine (mg/dl)	0.83 (0.72-1.09)	0.73 (0.64-0.99)	1.10 (0.83-1.16)	0.81 (0.74-1.06)	0.053
Donor					
DBD	26 (66.7)	7 (53.8)	5 (71.4)	14 (73.7)	0.483
DCD	10 (25.6)	6 (46.2)	1 (14.3)	3 (15.8)	0.116
LD	2 (5.1)	0 (0.0)	1 (14.3)	1 (5.3)	0.385
CIT (minutes)	463 (398-544)	415 (404-531)	537 (463-556)	479 (380-540)	0.427

[a] Patients are classified according to the most intense labelling; [b] Kruskal-Wallis tests for quantitative variables and X^2 tests for nominal variables. Abbreviations: CIT, cold ischaemia time; DBD, donor after brain death; DCD, donor after cardiac death; IQR, interquartile range; LD, live donor; LT, liver transplantation
